# Frequency of synaptic antigen-specific CD4^+^ T cells in dementia is age-dependent but not correlated with cognitive impairment

**DOI:** 10.1186/s12979-025-00516-w

**Published:** 2025-06-19

**Authors:** Julius Hoffmann, Marie-Luise Machule, Jakob Kreye, Laura Stöffler, Péter Körtvelyessy, Maria Buthut, Rosa Rößling, Petra Bacher, Alexander Scheffold, Harald Prüss

**Affiliations:** 1 German Center for Neurodegenerative Diseases (DZNE) Berlin, c/o Charité – Universitätsmedizin Berlin, Charitéplatz 1, Berlin, 10117 Germany; 2 Department of Neurology and Experimental Neurology, Freie Universität Berlin, Humboldt-Universität Zu Berlin, Berlin Institute of Health, Charitéplatz 1, Berlin, 10117 Germany; 3 Department of of Pediatric Neurology, Freie Universität Berlin, Humboldt-Universität Zu Berlin, Berlin Institute of Health, Berlin, Germany; 4 German Center for Neurodegenerative Diseases (DZNE) Magdeburg, Magdeburg, Germany; 5 Labor Berlin, Innovations, Berlin, Germany; 6 Berlin Institute of Health at Charité – Universitätsmedizin Berlin, Charitéplatz 1, Berlin, 10117 Germany; 7 Institute of Immunology, Christian-Albrecht-University of Kiel, Kiel, Germany; 8 Institute of Clinical Molecular Biology, Christian-Albrecht-University of Kiel, Kiel, Germany

**Keywords:** Synaptic autoantigens, Immune senescence, T cells, Dementia, NMDA receptor, LGI1, MGluR5

## Abstract

Neurodegenerative dementias including Alzheimer disease severely impair cognitive and social abilities and are a major cause of mortality with no causal treatment yet. Autoimmune mechanisms have been increasingly considered to contribute to disease progression, e.g. by enhancing protein misfolding or pro-inflammatory immune responses. Understanding this contribution may lead to novel treatment options beyond removing neurodegeneration-associated proteins. We hypothesized that CD4^+^ T_H_ cells against synaptic proteins may play a role in dementia, given the profound changes of synaptic proteins in the disease. We investigated T_H_ cell frequencies and phenotypes after antigen-reactive T cell enrichment (ARTE) using three important synaptic antigens known to play a role in cognitive function, N-Methyl-D-Aspartate receptor (NMDAR), Leucine-rich, glioma inactivated 1 (LGI1) and metabotropic glutamate receptor 5 (mGluR5). Our data revealed that synaptic autoantigen-specific T_H_ cells occurred in all cohorts and were similarly frequent in patients with dementia and sex- and age-matched controls. However, they were significantly reduced compared to young healthy subjects, indicating strong age-related effects (‘immune senescence’). Compared to the ubiquitously available Candida albicans antigen, synaptic autoantigen-specific T_H_ cell responses were strongly driven by IFNγ-producing T cells, expression of which markedly decreased with age. Patients with dementia had significantly less IL-17-producing synaptic autoantigen-specific T_H_ cells than aged healthy controls. This first direct ex vivo quantitative and qualitative analysis of circulating T cells autoreactive to three synaptic autoantigens in dementia shows no correlation with cognitive impairment. It suggests that synaptic autoantigen-specific T_H_ cells decline with age and are not a major driver of dementia development.

**Supplementary Information:**

The online version contains supplementary material available at 10.1186/s12979-025-00516-w.

## Background

Neurodegenerative dementias, including its most common form Alzheimer disease (AD), increase with age, will affect nearly 150 million people worldwide in 2050 [1] and are among the top ten causes of death [2]. The clinical symptoms, such as decreased cognitive, emotional and social abilities, but also difficulties with language, thinking and motivation, severely impair the activities of daily living. Causal therapies targeting degeneration of the brain are still missing [2, 3], even though Lecanemab and Donanemab, β-amyloid targeting therapeutic antibodies just recently demonstrated slowing of clinical progression in AD [4, 5]. One reason likely relates to the still limited understanding of disease pathology and the focus on removing disease-associated proteins such as β-amyloid, tau or α-synuclein [6].

For a few years, autoimmune mechanisms have been increasingly analyzed for their contribution to trigger protein misfolding and to drive disease progression in neurodegenerative diseases [7–10], potentially involving CNS-resident immune cells [11] and both, the innate and adaptive immune system [12, 13]. CD4^+^ T helper (T_H_) cells may play a particularly important role given their function to activate cytotoxic CD8^+^ T cells and stimulate the development of antibody-secreting cells. They can patrol the cerebrospinal fluid and were detected in the brains of patients with Alzheimer disease where they recognized β-amyloid antigens [14–19]. Likewise, T_H_ cells from patients with Parkinson disease can initiate a pro-inflammatory immune response after recognition of α-synuclein [20]. Although it is plausible that cytokine secretion by effector T_H_ cells may cause chronic neuroinflammation and thus facilitate neurodegeneration [21], the exact contribution of the immune system to the pathology of neurodegenerative dementias has yet to be clarified.

We hypothesized that not only T_H_ cells against neurodegeneration-associated proteins (such as β-amyloid) may play a role in dementia, but also T_H_ cells against synaptic proteins, given the profound changes of synaptic proteins at early stages during the development of cognitive impairment [22, 23]. We therefore investigated T_H_ cell frequencies, secretory activity and differentiation with flow cytometry after antigen-reactive T cell enrichment (ARTE) [24]. For this, three synaptic antigens were selected based on their molecular key role in cognitive function and for being established targets in antibody-mediated inflammatory brain diseases with predominant amnesia and cognitive impairment [25, 26]: the N-Methyl-D-Aspartate receptor (NMDAR), Leucine-rich, glioma inactivated 1 (LGI1) and the metabotropic glutamate receptor 5 (mGluR5). More specifically, the glutamatergic ion channel NMDAR is highly expressed in the hippocampus and involved in neuroplastic processes such as learning and memory formation [27]. Disruption of the trans-synaptic protein LGI1 impairs synaptic expression of multiple ion channels and causes dysfunctional synaptic excitability [28, 29]. The mGluR5 can mediate intracellular neuronal toxicity of soluble β-amyloid [30, 31], and hippocampal mGluR5 has been found to be reduced in early-stage AD [32]. At the clinical level, anti-NMDAR, LGI1 and mGluR5 autoantibodies were strongly associated with cognitive impairment [33, 34], AD-like phenotypes [35, 36] and profound amnesia [37], respectively.

We therefore analyzed the synaptic antigen-specific CD4^+^ T cells targeting NMDAR, LGI1 and mGluR5, aiming to assess their potential neuroimmunological contribution to cognitive impairment, also considering age-related changes of the immune system such as accumulation of memory T_H_ cells against synaptic antigens.

## Methods

### Cohorts

All clinical investigations were conducted according to Declaration of Helsinki principles. Written informed consent was received from participants at the outpatient memory clinic, Department of Neurology, Charité – Universitätsmedizin Berlin, prior to inclusion into the study. All analyses were approved by the Charité Institutional Review Board. Participants included (*1*) 27 patients with cognitive impairment, (*2*) 21 age- and sex-matched healthy controls and (*3*) 25 sex-matched young healthy controls (Table 1). Patients underwent screening of cognitive impairment (Mini-Mental State Examination; MMSE) that was performed by the same researcher. Dementia diagnoses were made according to established guidelines [38] and included AD (*n* = 14), *n *frontotemporal dementia (FTD, *n* = 7), vascular dementia (*n* = 4), amnestic mild cognitive impairment due to AD (MCI, *n* = 2). Participants with active malignancies were excluded from the screening. Healthy controls underwent the same neuropsychological screening and did not show cognitive impairment.

### Proteins for T cell stimulation

For the stimulation of peripheral blood mononuclear cells (PBMCs) we acquired NMDAR subunit NR1 (MyBiosource 964,741), LGI1 (MyBiosource 1,378,533) and mGluR5 (MyBiosource 960,840), recombinantly expressed in E.coli. According to the manufacturer, all samples showed a purity of at least 85%. Lysate of *Candida albicans* (C. alb., Greer Laboratories Cat#XPM15D3 A5) was used as positive control, a naturally occurring yeast fungus found on nasopharyngeal, intestine and urogenital mucosa. Dilution buffer 10 mM Tris–HCl (with 1 mM EDTA and 20% glycerol) served as negative control. All following steps were conducted the same day by the same researcher.

### Antigen-reactive T cell enrichment (ARTE)

PBMCs were isolated from venous blood samples (approximately 50 ml) collected in BD Vacutainer® CPT™ between 8:00–12:00 AM to prevent circadian fluctuations. The obtained cells were washed in PBS, quantified and plated in RPMI containing 5% human AB-Serum (Sigma-Aldrich, H4522). Cells were brought to a final concentration of 1 × 10^7^ cells per ml, plated in a 12-well plate with 1 ml volume per well and incubated at 5% CO_2_ and 37 °C overnight.

The enrichment of autoantigen-specific T cells was performed as previously described [24]. In brief, every 10^7^ PBMCs were incubated with 10 µg autoantigen (NR1, LGI1 or mGluR5) or 10 µl negative control (glycerol buffer, MyBiosource) and 20 µg *Candida*. 1 µg anti-CD40 antibodies (Miltenyi, 130–094–133, 100 µg/ml) was added to each stimulation to prevent interaction between CD40 and CD154 and subsequent internalization of CD154. After 5 h of incubation at 5% CO_2_ and 37 °C, 1 µg/ml Brefeldin A (Sigma-Aldrich, B6542) was added to inhibit the secretion of activation markers. After another 2 h, antigen-specific T_H_ cells were isolated using CD154-dependent magnetic activated cell sorting (MACS®) with the anti-CD154 Microbead Kit human (Miltenyi, 130–092–658) and MS columns (Miltenyi, 130–042–201).

**Fig. 1:**
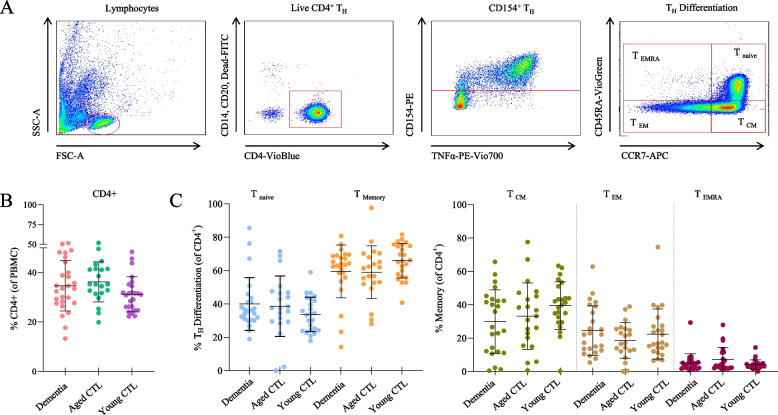
Normal CD4^+^ T_H_ cell frequencies in dementia patients and healthy controls. **A** Flow cytometry gating strategy used in this project. Lymphocytes were selected from PBMCs, gated for live CD4^+^ T_H_ cells and then for CD154^+^ T_H_ cells followed by differentiation of memory and naïve T cell subsets. **B** Without antigen stimulation, PBMCs showed similar frequencies of CD4^+^ T_H_ cells in all cohorts. **C** Naïve T cells and memory T cells (including memory T subsets) did not differ between all three cohorts in unstimulated condition

To determine the ratio of activated CD154^+^ T_H_ cells within all T_H_ cells as well as the frequency of T_H_ cells within all PBMCs, one sample per participant remained unstimulated (Fig. 1; original fraction, ORI). To avoid bias and reduce batch effects, we strictly adhered to technical protocols, always run positive and negative controls in parallel, measured patients and controls in parallel, and carefully assessed and adjusted FACS gates if needed.

### Cell staining and Fluorescence activated cell scanning (FACS)

Extracellular markers were stained with antibodies targeting CD14 (FITC, Miltenyi, TÜK4, 130–113–146, 1:50), CD20 (FITC, Miltenyi, LT20, 130–113–373, 1:50), CD4 (VIT4, VioBlue, Miltenyi, 130–113–219, 1:50), CD45RA (VioGreen, Miltenyi, REA562, 130–113–931, 1:50) and CCR7 (APC, Miltenyi, REA546, 130–120–466, 1:50). Furthermore, a viability staining was applied (488/520 FITC, Miltenyi, 130–109–812).

Following cell permeabilization and fixation (Miltenyi inside stain kit, Miltenyi 130–090–477), intracellular epitopes were stained with antibodies targeting CD154 (PE, Miltenyi, 5 C8, 130–113–607, 1:50), Interferon gamma (IFNγ, PerCP-Cy5.5, Biolegend, 4S.B3, 502,526, 1:100), tumor necrosis factor alpha (TNFα, PE-Vio770, Miltenyi, cA2, 130–120–492, 1:50) and Interleukin 17 (IL-17, APC-Vio770, Miltenyi, CZ8-23G1, 130–096–656, 1:10). FACS analysis was conducted immediately after cell staining. Measurements were performed using a BD FACS Canto™ II (BD, 338,962) and analyzed with FlowJo™ v10 (FlowJoLLC, Fig. 1).

### Statistical analysis

GraphPad Prism 8 software (GraphPad Software, Inc.) was used for statistical analyses. Prior to data analysis, background reactivity (CD154^+^ TH in negative control) was subtracted. Shapiro–Wilk and D’Agostino Pearson tests were applied to evaluate normal distribution. To compare two normally distributed populations a two-sided t-test for independent samples was used. More than two normally distributed populations were compared using a one-way ANOVA and a Tukey’s or Šídák’s post-hoc test for multiple comparisons. Linear regression analysis was performed and evaluated with regards to p, F and R2 values. The level of significance was defined as *p* < 0.05. Each population is presented with its individual values, their mean and standard deviation.

## Results

### Study participants

Study cohorts included patients with cognitive impairment (“*dementia*”, *n* = 27) and age- and sex-matched healthy controls (“*Aged CTL*”, *n* = 21). As the immune system underlies age-dependent changes, such as reduction in the T cell repertoire, another sex-matched control cohort of young subjects was recruited (“*Young CTL*”, *n* = 25). The mean age was 72.5 ± 9.5 years [± SD] in patients (15 M, 12 F), 74.6 ± 5.6 years in aged controls (10 M, 11 F) and 24.6 ± 3.8 years in young controls (12 M, 13 F). Additional clinical and treatment data are provided in Table 1.

**Table 1:** Demographic and Clinical Characteristics of Patients and Controls

Cohort (n)	Mean Age, (SD)	Gender ratio (m:f)	Mean MMSE score (SD)	Subtype of dementia	Comorbidities	Disease modifying/Immunosuppressive medication
Aged, Dementia (27)	72.5 (9.5)	15:12	21.0 (8.2)	4 × AD, early onset	1 × PLMD 1 × vitiligo	3 × ChEI
				10 × AD, late onset	2 × hypothyroidism 2 × hypercholesterinemia 1 × depression 1 × prostatic hyperplasia	8 × ChEI 1 × memantine
				7 × FTD	1 × prostate cancer (cured) 1 × arterial hypertension 1 × depression 1 × bronchial asthma	1 × benzothiazole 1 × hydroxychloroquine
				4 × vascular dementia	2 × arterial hypertension 1 × migraine 1 × diabetes (type 2) 1 × restless legs syndrome	1 × ChEI
				2 × MCI	2 × arterial hypertension 1 × bronchial asthma 1 × diabetes (type 2) 1 × Hashimoto’s thyreoiditis	1 × ChEI 1 × dopamine agonist
Aged, Healthy (21)	74.6 (5.6)	10:11	29.0 (1.4)		3 × diabetes (type 2) 1 × hyperuricemia 1 × hypercholesterinemia 1 × cardiac insufficiency	None
Young, Healthy (25)	24.6 (3.8)	12:13	30 (0)		1 × Marfan syndrome	None

*ChEI* cholinesterase inhibitors, *PLMD* Periodic Limb Movement disorder

### Similar CD4^+^TH cell frequencies in the blood of patients and controls

Dementia patients and both control cohorts had similar frequencies of CD4^+^ T_H_ cells in PBMCs without antigen stimulation (Fig. 1B). Likewise, the composition of naïve T cells and memory T cell subsets was not different between all three cohorts (Fig. 1C). Within the memory T cell pool, the highest fractions consisted of T_CM_ cells, followed by T_EM_ cells and T_EMRA_ cells, representing a typical T cell composition in the peripheral blood (Fig. 1D).

### Age-dependent frequency of antigen-specific TH cells reacting with Candida albicans and synaptic proteins

**Fig. 2:**
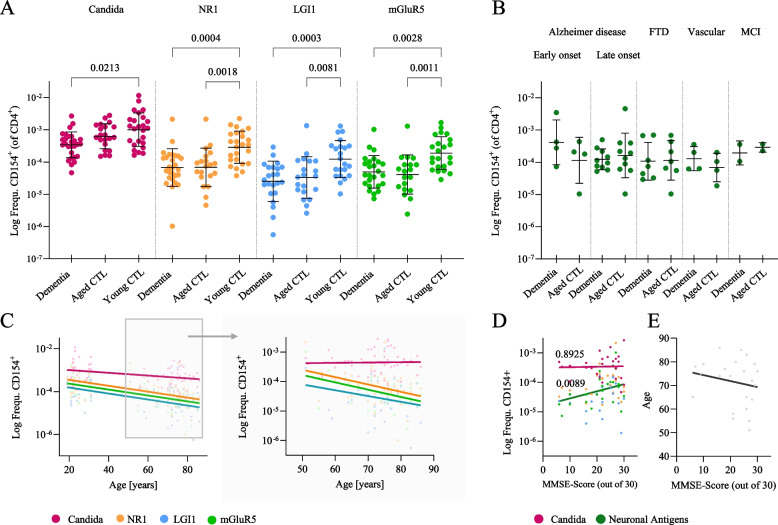
CD154^+^ T_H_ cells targeting neuronal antigens decrease with age. **A** Frequencies of CD154^+^ T_H_ cells after stimulation with the respective antigen (Candida, NR1, LGI1, mGluR5) were similar in dementia patients and aged controls, but markedly lower compared to young controls. **B** No differences between CD154^+^ T_H_ cell frequencies after stimulation with neuronal surface antigens among different dementia subgroups. **C** Significant age-dependent decrease of neuronal antigen-specific CD154^+^ T_H_ cells. The reduction was particularly obvious in the aged group, where no decrease was observed following stimulation with *Candida albicans* (right). **D** Regression analyses illustrating the relationship between CD154^+^ T_H_ cell frequencies and MMSE score, displaying a significant decrease of neuronal antigen-specific, but not *Candida*-specific CD154^+^ T_H_ cells in patients with reduced MMSE scores. **E** In regression analyses between MMSE scores and age, MMSE scores did not significantly correlate with age

We next determined the T cell response against the ubiquitously present *Candida albicans*, which is known to generate strong T cell responses in the majority of donors. T_H_ cell frequencies were not significantly different between dementia patients and aged controls, but lower than in young controls (Fig. 2A), suggesting an age-dependent loss of reactivity [39]. Stimulation with NMDAR, LGI1 and mGluR5 proteins resulted in enrichment of CD154-expressing T cells, demonstrating the presence of specific T_H_ cells against all three synaptic antigens in patients and controls. The frequencies followed a similar pattern compared to *Candida*-specific T cells, being more frequent in young controls compared to aged controls and dementia patients (NMDAR > mGluR5 > LGI1, Fig. 2A). As expected, *Candida*-specific T_H_ cells are generally more frequent than synaptic antigen-specific cells [40]. The mean frequencies of T_H_ cells against all three antigens were similar between the different dementia subtypes (Fig. 2B).

While the frequency of *Candida*-specific T_H_ cells was lower in the aged compared to the young cohort (Fig. 2C, left), the frequency remained stable when looking only into the subgroup of aged participants (dementia patients and aged controls) between 51–86 years (Fig. 2C, right). In contrast, synaptic antigen-specific T_H_ cells in this subgroup further decreased with higher age. In a linear regression analysis, significant decreases were seen for all three synaptic antigens with a similar slope (Fig. 2C). Interestingly, T_H_ cell frequencies against neuronal antigens, but not against *Candida*, significantly decreased with worsening of cognitive impairment based on MMSE scoring (Fig. 2D). However, this effect depended on the age-related reduction in MMSE scores (Fig. 2E) according to multiple regression analyses (data not shown; for *C. albicans*: T_H_ cell frequency vs. MMSE *p* = 0.0962; T_H_ cell frequency vs. age: *p* = 0.4359; for neuronal antigens: T_H_ cell frequency vs. MMSE: *p* = 0.1516; T_H_ cell frequency vs. age: *p* = 0.0009).

### T_memory_cells were more frequent in young controls after synaptic antigen stimulation

**Fig. 3:**
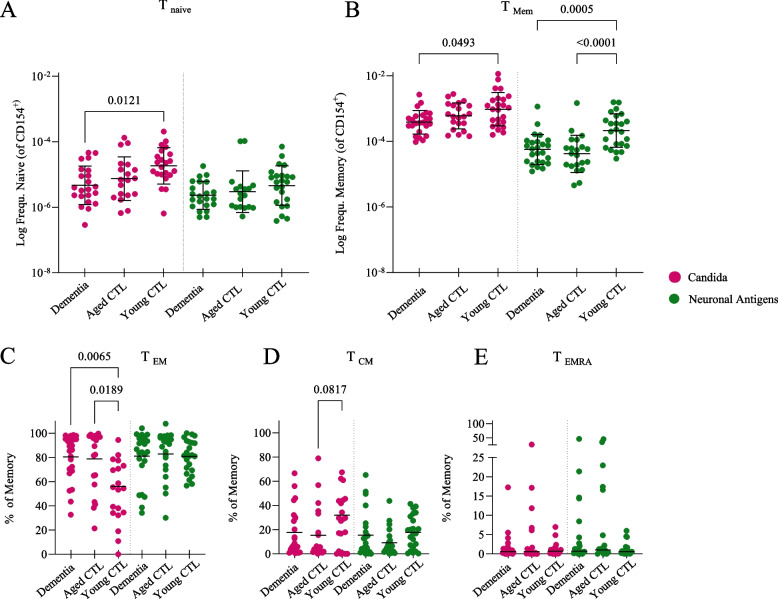
T_memory_ cells were most strongly linked to the age-dependent reduction in T_H_ cell frequency after synaptic antigen stimulation. **A** Naïve T_H_ cells against synaptic antigens were equally frequent in all cohorts, in contrast to increased frequencies in the *Candida*-stimulated group of young controls. **B** Memory T_H_ cells were significantly more frequent in young controls in both, the *Candida*-stimulated and synaptic antigen-stimulated groups. **C**-**E** Within the memory compartment, T_EM_ were more frequent than T_CM_ and T_EMRA_ cells. Within those subsets, no differences were found between cohorts after stimulation with neuronal antigens

Given the similar frequencies of T_H_ cells responding to NMDAR, LGI1 and mGluR5 protein together with the identical age-related decline trajectories, for the following analyses we pooled data using mean frequencies from all three antigens. After stimulation with synaptic antigens, naïve CD154-expressing T cells were equally frequent in all cohorts, in contrast to increased frequencies in the *Candida*-stimulated group (Fig. 3A, Suppl. Figure 1 A). The memory pool, however, showed markedly increased frequencies in young controls compared to dementia patients and aged controls in both, the *Candida*-stimulated and synaptic antigen-stimulated groups (Fig. 3B). In young donors the response to *Candida* displayed a decreased T_EM_ (Fig. 3C) versus and increased T_CM_ response (Fig. 3D), T_EMRA_ cells were not different (Fig. 3E; Suppl. Figure 1 shows frequencies for individual synaptic antigens).

### Variable production of inflammatory cytokines in CD154^+^T cells

**Fig. 4:**
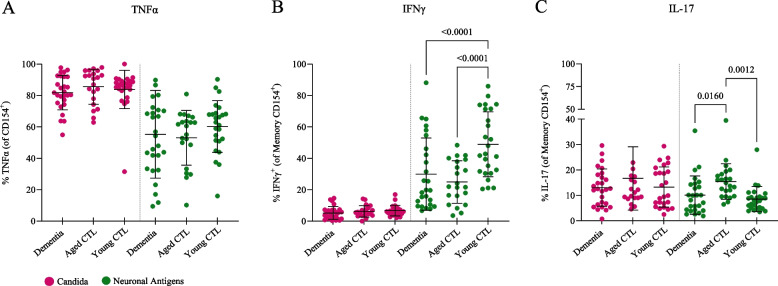
Differential cytokine profiles in CD154^+^ T_H_ cells. **A** TNFα was the main inflammatory cytokine in *Candida*-specific T_H_ cells. Lower frequencies were observed in T_H_ cells after stimulation with neuronal antigens. **B** IFNγ production predominated in synaptic antigen-specific CD4^+^ T_H_ cells, in particular in young healthy controls with nearly twice as much IFNγ production compared to aged controls and dementia patients. **C** IL-17 production after stimulation with synaptic antigens was significantly increased in healthy aged subjects compared to dementia patients and young controls

We next determined whether antigen-activated T cells from dementia patients differed in the expression levels of inflammatory cytokines. While TNFα was the main cytokine in all cohorts after *Candida* stimulation, it was markedly less produced after stimulation with synaptic antigens (Fig. 4A). In contrast, IFNγ production predominated in synaptic-antigen-specific CD4^+^ T cells, while almost no *Candida*-specific T_H_ cells were IFNγ-positive (Fig. 4B). Similar to the T_H_ cell frequencies, the IFNγ expression in memory cells was clearly age-specific (but not dementia-specific) with nearly twice as much IFNγ production in young controls compared to aged controls and dementia patients (Fig. 4B).

A different pattern was observed for the production of IL-17 in antigen-reactive CD154^+^ T_H_ cells. IL-17 expression after stimulation with the ubiquitous *Candida* antigen showed an equal response between cohorts at low frequencies (Fig. 4C). In contrast, IL-17 production after stimulation with synaptic antigens was significantly increased in healthy aged subjects (Fig. 4C) compared to dementia patients and young controls (Suppl. Figure 2 shows percentages of individual synaptic antigens).

## Discussion

The present study provides the first direct ex vivo quantitative and qualitative analysis of circulating T cells autoreactive to three important synaptic autoantigens in patients with dementia. Our data reveal several unexpected findings: (1) synaptic autoantigen-specific memory T_H_ cells were detected in all cohorts, with NR1 being the most common antigen; (2) T_H_ cells specific for synaptic autoantigens were similarly frequent in patients with dementia and sex- and age-matched controls, however, were significantly reduced compared to young healthy subjects, indicating strong age-related effects; (3) compared to *Candida* antigen, synaptic autoantigen-specific T_H_ cell responses are strongly driven by IFNγ-producing T cells and decrease with age; (4) aged donors displayed increased frequencies of Th17 cells within the synaptic autoantigen-specific CD154^+^ T_H_ cells as compared to young donors, and these increased Th17 cell levels were absent in dementia patients.

The data collectively show that, while all participants in this study had T_H_ cells reacting to all examined synaptic autoantigens, neither their frequency nor the type of neuronal autoantigen significantly correlated with cognitive impairment, suggesting that the autoantigen-specific T_H_ cell response is not a major driver of dementia development.

As CD8^+^ CTLs do not generally express CD154, they were not measured in this experimental setup. Therefore, no direct statement can be made about their potential role in this respect. However, it is generally assumed that CD8^+^ T cell responses require the help of CD4^+^ T_H_ cells [41]. Therefore, we assume that the absence of a strong CD4^+^ T_H_ cell response to the measured neuronal antigens and the lack of a correlation with dementia argue against a strong CD8^+^ T cell reactivity.

The proportions of unstimulated CD4^+^ T_H_ cell subsets in the PBMCs of dementia patients were similar to controls and consistent with published reference ranges in healthy individuals [42], including high levels of naïve T_H_ cells persisting into old age [43]. Thus, dementia patients did not have a confounding general immunocompromised condition, which could otherwise have affected the response to autoantigen stimulation.

After antigen stimulation, synaptic autoantigen-specific T_H_ cells were much less frequent than those reacting against the foreign *Candida* antigen, in line with current concepts of antigen-specific T cells [40]. Interestingly, T_H_ cells responding to (synaptic) autoantigens were dominated by T_EM_ cells in all donor groups suggesting that autoreactive T_H_ cells preferentially accumulate within this subset. In contrast, the response to *Candida* was dominated by T_EM_ cells only in the aged donor groups, whereas T_EM_ cells were decreased in young donors, which instead displayed a tendency towards increased T_CM_ cells. We could confirm that *Candida*-specific T cells almost exclusively utilized TNFα and some IL-17 [44, 45], while synaptic autoantigen-specific T cells secreted TNFα and to a comparably high degree IFNγ, but also IL-17. It is unclear whether this signature reflects autoimmunity and CNS-inflammation [46, 47] and can support trans-endothelial migration of CD4^+^ T_H_ across the blood–brain barrier [48]. Given the limited set of cytokines examined here, we cannot comment on different cytokines, such as IL-4 or IL-5.

In contrast to unstimulated T_H_ cells, the frequency of synaptic autoantigen-specific CD4^+^ T cells was strongly age-dependent with approximately tenfold reduction of frequency between 25 and 75 years of age. It is likely unrelated to the natural decrease in TCR diversity beyond the age of 60, which mainly reflects a decrease in naïve T_H_cells [49–51]. Future research will determine whether the concept of ‘immune senescence’, i.e. the gradual deterioration of the immune system with age, also encompasses T_H_ cells against synaptic antigens, as suggested by our findings.

We cannot comment on the proportion of synaptic antigen-specific T_reg_ and their contribution to the T cell pool, as T_reg_ cells do not express CD154 after 7 h of antigen stimulation [52] and are therefore not antigen-specifically enriched. A possible future approach for the detection of autoreactive T_reg_ cells might be a selection based on CD137 expression [53].

The age-related decrease of antigen-specific T_H_ cells was paralleled by a marked reduction of IFNγ-producing synaptic autoantigen-specific T_H_ cells with age, which was surprising given that most studies reported increased IFNγ secretion in aged individuals [54]. The pattern was also clearly different from the foreign *Candida* antigen. Further studies will be required to determine the function and effects of high IFNγ production by antigen-specific T_H_ cells in young compared to aged people, which may range from pro-inflammatory effects to T_reg_ induction.

Traditional views suggest that high IL-17 levels preferably promote neuroinflammation, autoimmunity and neurodegeneration in humans and rodent models, although recent data indicate more nuanced tissue-dependent functions including protective roles [55]. Following the hypothesis of IL-17-stimulated (neuro)inflammation, we assumed that increased IL-17-producing neuronal autoantigen-specific T cells can amplify inflammation in the brain and thus lead to autoimmunity-driven cognitive impairment. However, dementia patients in our cohorts had significantly lower frequencies of IL-17-producing synaptic autoantigen-specific T_H_ cells, which corresponds to the observation that CSF levels of IL-17 were negatively correlated with disease progression in AD [56]. Thus, future work should include the generation of T cells lines from dementia patients and control subjects to better understand their detailed role in neuroinflammation. The potentially protective function of synaptic autoantigen-specific IL-17-producing T_H_ cells may be of particular interest, given that the IL-17 pathway contains druggable targets for AD.

## Supplementary Information

Supplementary Material 1. Suppl. Figure 1: Profiles of CD154+ TH cell subgroups following antigen stimulation with Candida albicans and the synaptic proteins NR1, LGI1 or mGluR5. (A-D) In all cohorts and irrespective of the antigen used for TH cell stimulation, TEM (B) were most frequent followed by TCM (C), naïve TH cells (A) and TEMRA (D). Suppl. Figure 2: Cytokine secretion in CD154+ TH cells following antigen stimulation with Candida albicans and the synaptic proteins NR1, LGI1 or mGluR5. (A) TNFα was the main cytokine in all cohorts, in particular in Candida-specific TH cells. (B) In contrast, IFNγ production predominated in synaptic antigen-specific CD4+ TH cells with highest levels in young healthy control subjects. (C) IL-17 production after stimulation with NR1 and mGluR5 was significantly increased in TH cells of healthy aged subjects compared to dementia patients and young controls.

## Data Availability

The datasets used and analysed during the current study are available from the corresponding author on reasonable request.

## Abbreviations

AD: Alzheimer disease
AMPA: α-Amino-3-hydroxy-5-methyl-4-isoxazolepropionic acid
BBB: Blood brain barrier
ChEI: Cholinesterase inhibitors
CTL: Cytotoxic T lymphocytes
FACS: Fluorescence activated cell scanning
FTD: Frontotemporal dementia
IFNγ: Interferon gamma
IL-17: Interleukin 17
LGI1: Leucine-rich, glioma inactivated 1
MACS: Magnetic cell sorting
MCI: Mild cognitive impairment
mGluR5: Metabotropic glutamate receptor 5
NMDA-R: N-Methyl D-Aspartate receptor
ORI: Original fraction
PBMC: Peripheral mononuclear blood cells
PLMD: Periodic Limb Movement disorder
T_CM_: Central memory T_H_ cell
TCR: T cell receptor
T_EM_: Effector memory T_H_ cell
T_EMRA_: Effector memory T_H_ cell re-expressing CD45RA
T_H_: T helper cell
TNFα: Tumor necrosis factor alpha
T_reg_: Regulatory T_H_ cell
