# Supplementary material for: Frequency of synaptic antigen-specific CD4+ T cells in dementia is age-dependent but not correlated with cognitive impairment
Source: Immun Ageing. 2025 Jun 19;22:23. doi: 10.1186/s12979-025-00516-w (PMC12178052; doi:10.1186/s12979-025-00516-w)
Supplement: Supplementary file 1 — Supplementary Material 1. Suppl. Figure 1: Profiles of CD154+ TH cell subgroups following antigen stimulation with Candida albicans and the synaptic proteins NR1, LGI1 or mGluR5. (A-D) In all cohorts and irrespective of the antigen used for TH cell stimulation, TEM (B) were most frequent followed by TCM (C), naïve TH cells (A) and TEMRA (D). Suppl. Figure 2: Cytokine secretion in CD154+ TH cells following antigen stimulation with Candida albicans and the synaptic proteins NR1, LGI1 or mGluR5. (A) TNFα was the main cytokine in all cohorts, in particular in Candida-specific TH cells. (B) In contrast, IFNγ production predominated in synaptic antigen-specific CD4+ TH cells with highest levels in young healthy control subjects. (C) IL-17 production after stimulation with NR1 and mGluR5 was significantly increased in TH cells of healthy aged subjects compared to dementia patients and young controls. [file 12979_2025_516_MOESM1_ESM.pptx]

## Slide 1
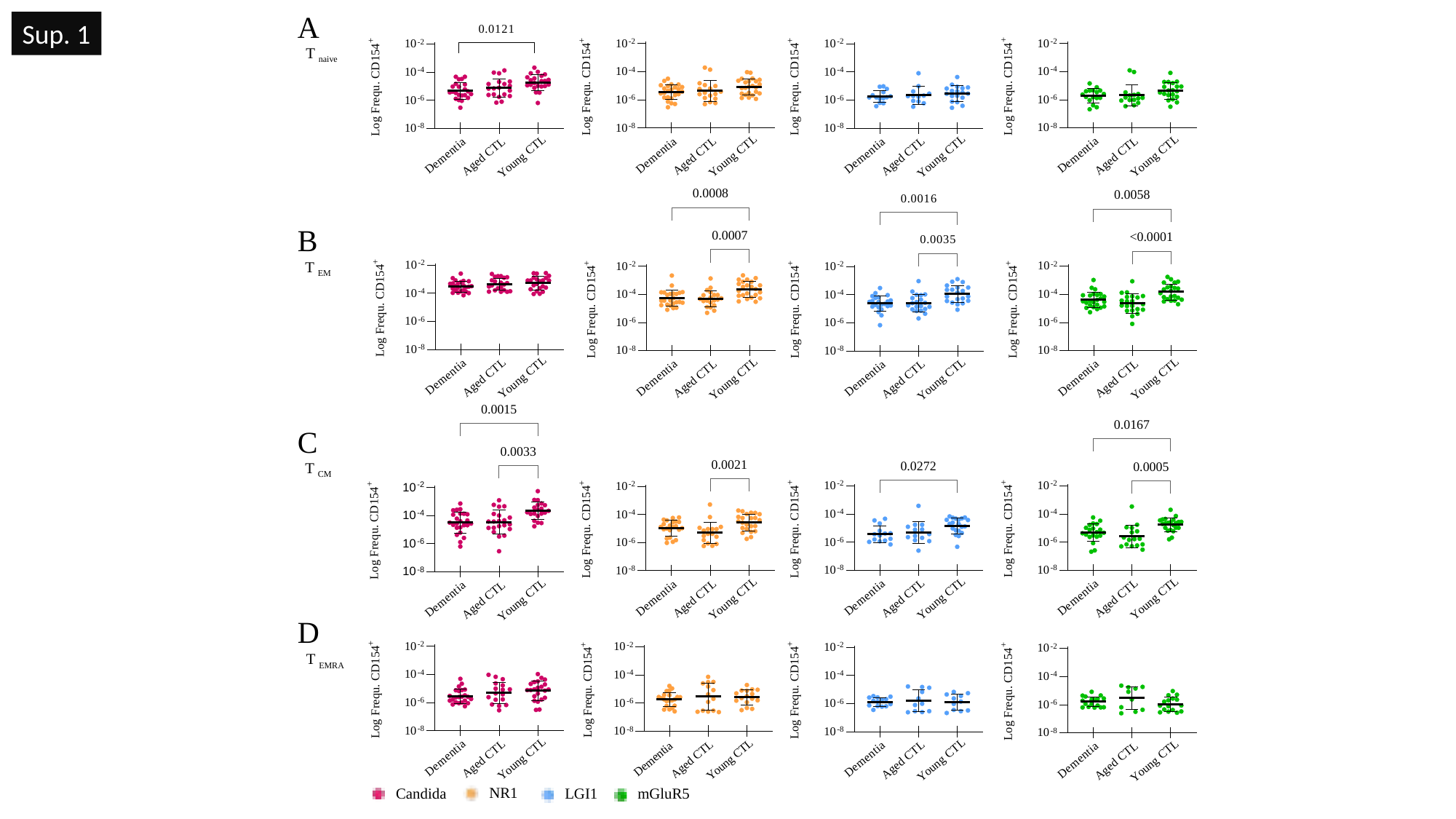

A
Sup. 1
T naive
B
T EM
C
T CM
D
T EMRA
NR1
LGI1
mGluR5
Candida

## Slide 2
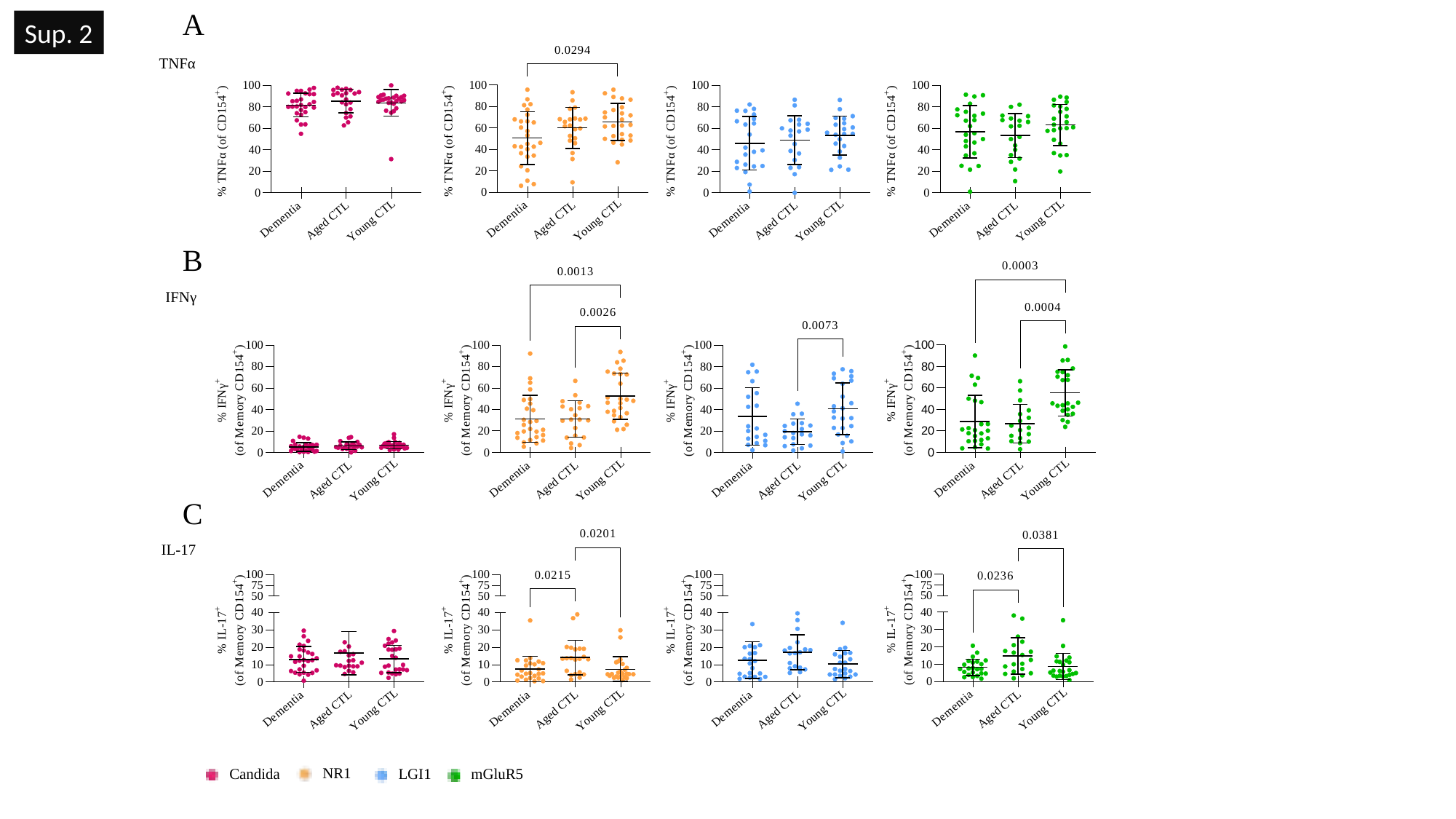

A
Sup. 2
TNFα
B
IFNγ
C
IL-17
NR1
LGI1
mGluR5
Candida
